# Solitary splenic metastasis from nasopharyngeal carcinoma: a case report and systematic review of the literature

**DOI:** 10.1186/s12957-016-0941-2

**Published:** 2016-07-15

**Authors:** Pietro Genova, Francesco Brunetti, Emilie Bequignon, Filippo Landi, Vincenzo Lizzi, Francesco Esposito, Cecile Charpy, Julien Calderaro, Daniel Azoulay, Nicola de’Angelis

**Affiliations:** Department of General and Oncological Surgery, Azienda Ospedaliera Universitaria Policlinico “Paolo Giaccone”, Via del Vespro 129, 90127, Palermo, PA Italy; Department of Digestive, Hepatobiliary Surgery and Liver Transplantation, Henri Mondor University Hospital, AP-HP, Université Paris Est - UPEC, Créteil, France; Department of Otorhinolaryngology and Head and Neck Surgery, Henri Mondor University Hospital, AP-HP, Université Paris Est - UPEC, Créteil, France; INSERM U955, Créteil, France; Department of Pathology, Henri Mondor University Hospital, AP-HP, Université Paris Est - UPEC, Créteil, France

**Keywords:** Splenic metastasis, Nasopharyngeal carcinoma, Systematic review

## Abstract

**Background:**

Solitary splenic metastases are a rare occurrence, and the nasopharyngeal carcinoma represents one of the most uncommon primary sources. The present study aimed to describe a rare case of a solitary single splenic metastasis from nasopharyngeal carcinoma and to assess the number of cases of isolated nasopharyngeal carcinoma metastases to the spleen reported in the literature.

**Main body:**

We describe the case of a 56-year-old man with a history of nasopharyngeal carcinoma and complete remission after chemo-radiotherapy. Three months after complete remission, positron emission tomography/computed tomography scan revealed a hypermetabolic splenic lesion without increased metabolic activity in other areas. After laparoscopic splenectomy, the pathology report confirmed a single splenic metastasis from undifferentiated carcinoma of the nasopharyngeal type. The postoperative period was uneventful. We also performed a systematic review of the literature using MEDLINE and Google Scholar databases. All articles reporting cases of splenic metastases from nasopharyngeal carcinoma, with or without histologic confirmation, were evaluated. The literature search yielded 15 relevant articles, which were very heterogeneous in their aims and methods and described only 25 cases of splenic metastases from nasopharyngeal carcinoma.

**Conclusion:**

The present review shows that solitary splenic metastases from nasopharyngeal carcinoma are a rare event, but it should be considered in patients presenting with splenic lesions at imaging and a history of primary or recurrent nasopharyngeal carcinoma. No evidence supports a negative impact of splenectomy in patients with solitary splenic metastasis from nasopharyngeal carcinoma.

## Background

Splenic metastases from non-hematologic malignancies are rare [1–4], but according to several studies, they can occur in cases of disseminated disease [5–7]. Their prevalence ranges from 0.6 % [3] to 7.1 % [8] in autopsy series of patients with cancer and from 1.1 % [3] to 3.4 % [9] in a series of patients who have undergone splenectomy.

The most frequent primary sources of splenic metastases from non-hematologic malignancies are breast, lung, ovarian, colorectal, and gastric adenocarcinomas, along with skin melanoma [3, 8, 10, 11]. By 2007, only 93 well-documented cases of solitary splenic metastases were reported [1], with colorectal and ovarian cancer being the most common sources and breast and skin melanomas the most uncommon [1, 12–15].

Among the uncommon primary sources of splenic metastases, there is the nasopharyngeal carcinoma (NPC) [3]. This type of tumor is sporadic in western countries (incidence: 0.5–2/100.000/year) and more frequent in certain endemic areas, such as southern China (incidence: 25/100.000/year, Hong Kong) [16, 17]. Intermediate-risk regions are the Middle East, southeastern Asia, northern Africa, and Alaska. NPC is usually unresectable at diagnosis, but it is more responsive to chemotherapy and radiotherapy than other cancers of the head and neck [18]. However, NPC is prone to early metastatic spread. Cervical lymph node metastases are present at diagnosis in 75–90 % of cases and are bilateral in more than 50 % of the cases [19–21]. A cervical lymph node advanced disease is linked to a higher risk of distant metastasis (33 % for N1, 70 % for N2/N3 at 10 years) and reduced survival. Distant metastases are present at diagnosis in 5–11 % of patients, and the most common sites are bone, lung and liver tissues. According to the 7th edition of the American Joint Committee Cancer Staging Manual [22], the 5-year survival rates by stage of NPC are 72 % for stage I, 64 % for stage II, 62 % for stage III, and 38 % for stage IV.

In the present study, we report the case of a patient with a complete remission (CR) of a NPC after chemo-radiotherapy, who underwent splenectomy for a solitary hypermetabolic splenic lesion detected at the 3-month follow-up. Additionally, we performed a systematic review of the literature with the aim of assessing the number and characteristics of the reported cases of splenic metastases from NCP.

## Case report

A 56-year-old Caucasian man, smoker, underwent an ENT examination for enlarged left cervical lymph node associated with left otalgia appearing 3 months earlier. At nasal endoscopy, a nasopharyngeal lesion extending from the left Rosenmüller fossa to the choanae was found, and biopsies were performed. The pathology report was conclusive for undifferentiated carcinoma of the nasopharyngeal type (UCNT). The cervical contrast-enhanced computed tomography (CT) showed enlarged jugular nodes bilaterally (largest sizes were 25 × 22 mm and 23 × 18 mm on the left side) with left jugular vein compression and signs of nodal necrosis. The thoracic scan detected an irregular nodule of 6 mm in the medial-basal segment of the right lung and non-specific micro-nodules in the left superior lobe. The abdominal scan showed no liver focal lesions or other organ involvement. A magnetic resonance imaging (MRI) was performed to assess more accurately the loco-regional extent of the tumor. It showed no intracranial extension and confirmed a skull base erosion (left basisphenoid inferior lysis) with thickening of the nasal fossae soft tissues (8 mm) at the level of the choanae. Cervical lymph node disease was confirmed at the IIB level on the right side and at the IB, II, III, and IV levels on the left side. A subsequent positron emission tomography (PET)/CT scan showed a high standardized uptake value (SUV) in the nasopharynx with involvement of the sphenoidal sinus and left greater wing. Serological tests for EBV were positive. The patient was classified as stage IVA (T4N2cM0) and treated with neoadjuvant chemotherapy followed by intensity modulated radiation radiotherapy (IMRT) and chemotherapy. The neoadjuvant chemotherapy consisted of 3 cycles of docetaxel, carboplatin and 5-fluorouracil, which were followed by IMRT (70 Gy on the nasopharynx and involved lymph nodes and prophylactic treatment with 56 Gy on the other nodal areas) and two cycles of cisplatin. A complete remission was obtained, with no evidence of disease on CT scan at the end of the treatment.

**Fig. 1:**
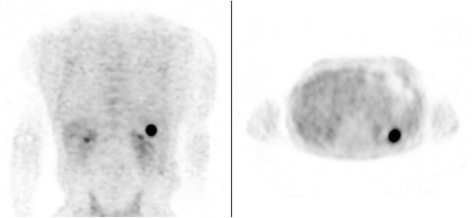
Imaging. After 3 months of complete remission following chemo-radiotherapy, a whole-body FDG-PET showed a splenic focal lesion with increased uptake

However, at 3 months from the end of treatment, the follow-up PET/CT scan detected a hypermetabolic nodule of the spleen (size: 24 × 17 mm) with SUV of 7. No other areas showed increased metabolic activity (Fig. 1). A biopsy of the splenic nodule was impossible to perform because of the nodule location and an incipient severe ischemic heart disease. The patient needed to be hospitalized and underwent coronary angioplasty and stenting. At 6 months, another PET/CT scan revealed increased size (33 × 26 mm) and metabolic activity (SUV 14) of the spleen nodule, without other suspicious lesions. The CT performed at 9 months confirmed that the pulmonary nodules had completely disappeared. No abdominal symptoms were observed. In accordance with the decision of a multidisciplinary team, the patient underwent standard laparoscopic splenectomy at both diagnostic and therapeutic aims.

The patient was positioned in a semi-lateral right decubitus position with a cushion placed under the right flank. Four ports were used. First, a 12-mm peri-umbilical optical port was placed with the Hasson technique [23, 24]. Then, the other three ports were visualized directly, and their locations were as follows: an epigastric 5-mm port slightly to the left of the median line, a 12-mm port, and another 5-mm port 4 cm below the left costal arch on anterior and middle axillary lines. A 30° laparoscope and bipolar radiofrequency device were used. The spleno-colic ligament was dissected to expose the lower pole of the spleen, and the gastro-splenic ligament was dissected to expose the splenic hilum. Careful dissection of the splenic artery and vein was performed. The hilar vessels were separated using a 30-mm vascular stapler, with the artery identified first. The diaphragmatic and posterior attachments of the spleen were then dissected, and the entire organ was removed through a Pfannenstiel incision. The operating time was 120 min.

**Fig. 2:**
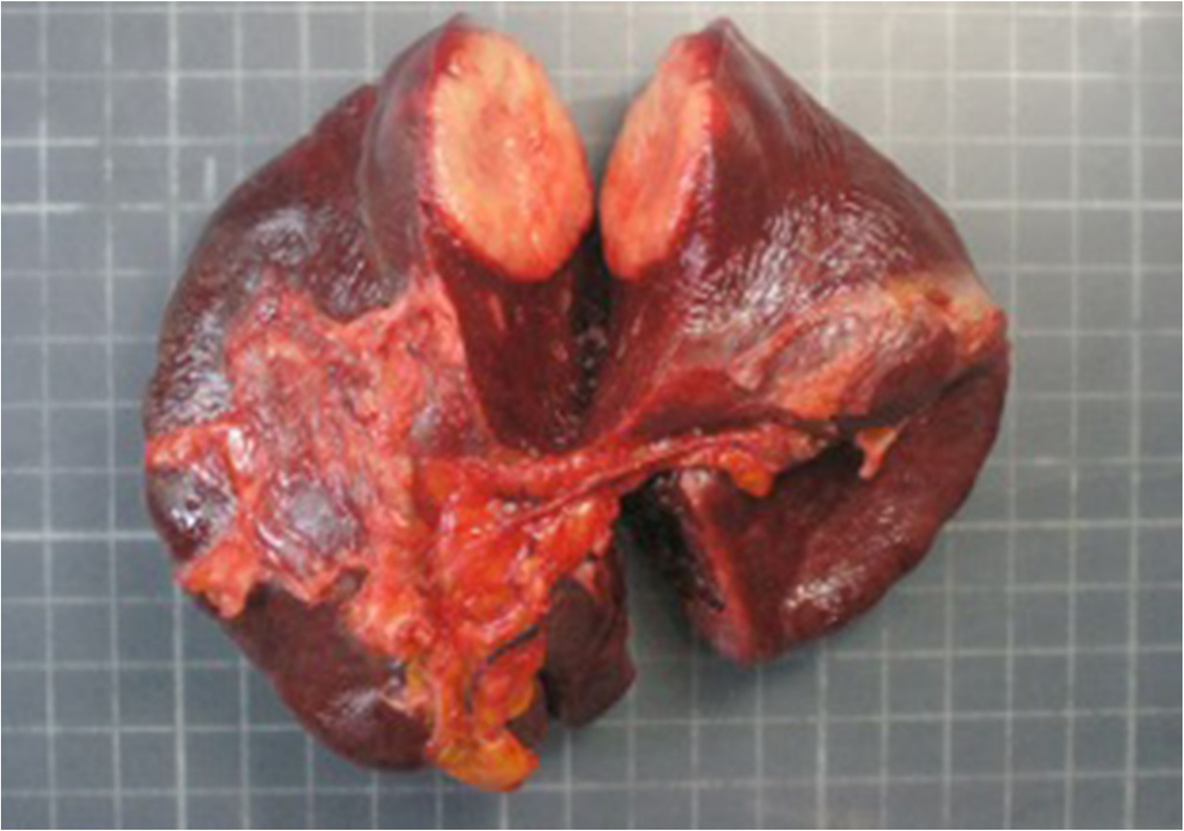
The pathologic specimen. The spleen weighed 236 g and measured 10.5 × 10.5 × 4.5 cm. The metastasis appeared as a gray, well-delimited, homogeneous, single nodule that was 38 × 35 mm in size and with 10 % of the section surface occupied by necrotic areas

**Fig. 3:**
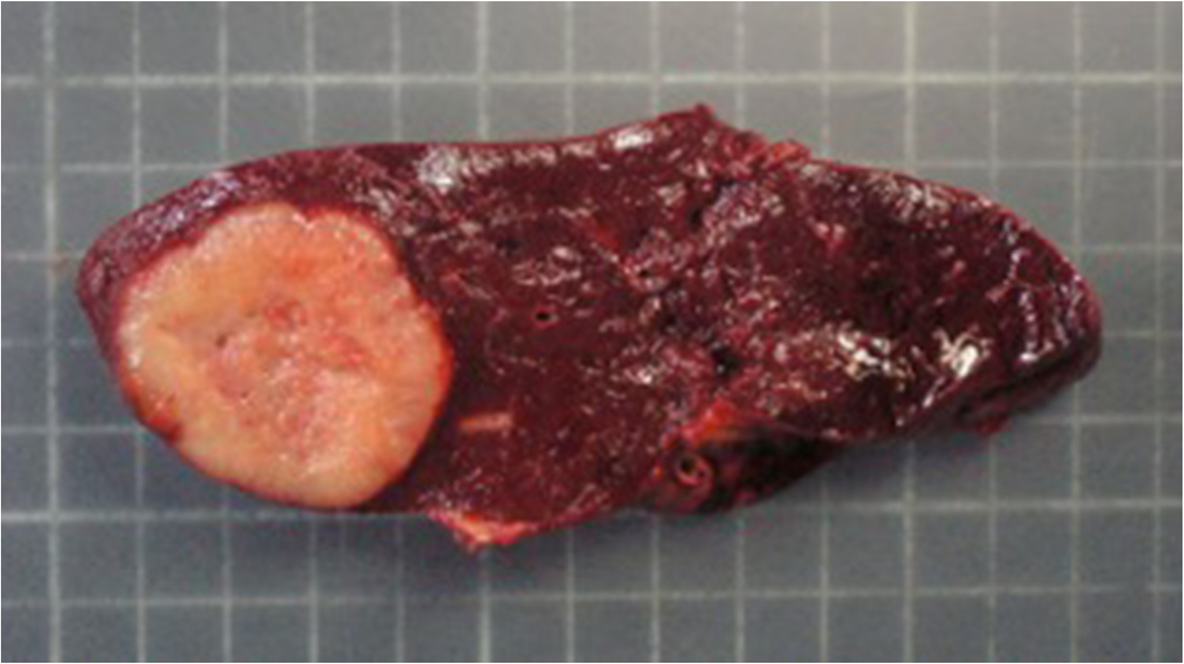
The pathologic specimen. The spleen weighed 236 g and measured 10.5 × 10.5 × 4.5 cm. The metastasis appeared as a gray, well-delimited, homogeneous, single nodule that was 38 × 35 mm in size and with 10 % of the section surface occupied by necrotic areas

The specimen weighed 236 g and measured 10.5 × 10.5 × 4.5 cm. The pathology report described a 38 × 35-mm sized single splenic nodule, which was well-delimited and homogeneous with morphological and immunophenotypical features consistent with a metastasis from UCNT (Figs. 2 and 3). An abdominal CT scan performed 6 days after the operation showed multiple thrombosis of the splenic vein and both right and left portal branches. Anticoagulation therapy was started. The patient was discharged 10 days after splenectomy. At 4 months of follow-up, the patient showed resolution of splenic and portal thrombosis and no evidence of recurrence. After discussion in a multidisciplinary meeting, no adjuvant treatments were performed.

## Systematic review of the literature

### Materials and methods

The methodological approach included the development of the selection criteria, definition of the search strategy, assessment of the study quality, and abstraction of the relevant data. The PRISMA statement checklist for reporting a systematic review was applied [25].

#### Study inclusion criteria

The study selection criteria were defined before starting the data collection to allow proper identification of the studies eligible for the analysis. All studies reporting distant metastasis from nasopharyngeal carcinoma or splenic metastases from different primary tumors were retrieved and checked for eligibility. The selection criteria included the following:

- Types of study: all types of original articles (including case report) with no limit of time.
- Types of participants: patients affected by NPC with splenic metastases detected by biopsy, imaging, or autopsy.

#### Literature search strategy

A literature search was performed with the following online databases: MEDLINE (through PubMed) and Google Scholar. A specific research equation was formulated using the following keywords and/or MeSH terms: spleen, metastasis, splenic metastasis, splenic neoplasms, nasopharyngeal, and nasopharyngeal neoplasms. In addition, reference lists from eligible studies and relevant review articles were crosschecked to identify additional studies. No time limitation was applied. Studies written in English, French, or Italian, and meeting the selection criteria were reviewed.

#### Study selection and quality assessment

The titles and abstracts of the retrieved studies were screened for relevance by two independent reviewers (PG, FB). Subsequently, a full-text analysis of the selected articles was carried out. Any disagreement between the two reviewers during the study selection process was resolved by discussion with a third reviewer (NdeA). The Grading of Recommendations Assessment Development and Evaluation (GRADE) system was used to grade the “body of evidence” emerging from this review [26].

#### Data extraction

All studies reporting cases of splenic metastasis from nasopharyngeal cancer were retrieved and included in this systematic review.

### Results

#### Literature search and selection

**Fig. 4:**
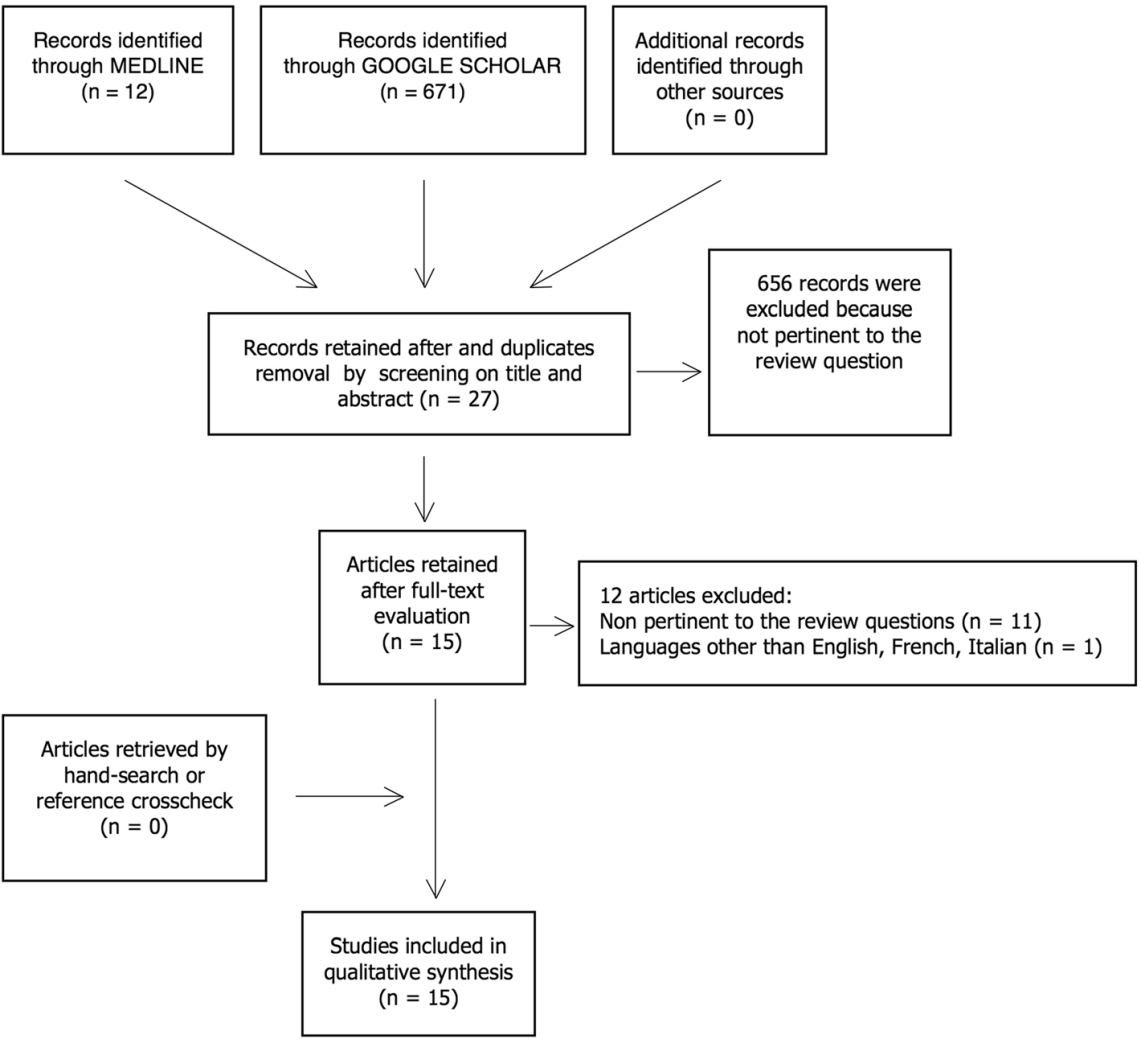
Flow chart of the search, selection, and inclusion processes for the systematic review of the literature. An example PubMed search equation: ((“spleen”[MeSH Terms] or “spleen”[all fields] or “splenic neoplasms”[MeSH terms] or “splenic metastasis”[all fields]) and (“neoplasm metastasis”[MeSH Terms] or (“neoplasm”[all fields] and “metastasis”[all fields]) or “neoplasm metastasis”[all fields] or “metastasis”[all fields])) and (“nasopharynx”[MeSH terms] or “nasopharyngeal neoplasms”[MeSH terms] or “nasopharynx”[all fields] or “nasopharyngeal”[all fields])

The preliminary literature search identified 682 articles. Of these, 655 articles were rejected because they were not pertinent to the review questions or duplicates, whereas 27 were retained after screening their titles and abstracts. At the full-text examination, 15 studies were selected. The manual search and the crosscheck of the reference lists did not yield other relevant articles. A flow chart illustrating the study identification and inclusion/exclusion processes is shown in Fig. 4.

#### Study characteristics

Among the 15 selected studies [3, 5, 27–39] reporting on splenic metastases from NPC, 13 were published between 2000 and 2015, one was published in 1989, and one was published in 1952. The included studies were very heterogeneous in their design, aims and methods. There were three case reports [27, 36, 38] and 12 case series [3, 5, 28-35, 37, 39]. The studies focused on NPC and the imaging techniques used for diagnosis (*n* = 5) [28, 33, 35, 37, 39], splenic metastases from solid tumors (*n* = 3) [3, 5, 31], experience of a single institution in NPC treatment (*n* = 2) [30, 34], use of serum markers in monitoring NPC systemic relapse (*n* = 1) [29], and chemotherapy in NPC patients (*n* = 1) [32]. Nine studies [3, 28, 29, 32–35, 38, 39] were performed in Asian populations, three in Western countries [5, 31, 37], one in the Middle East [27], one in Africa [30], and one in India [36]. Overall, 25 cases of splenic metastases from NPC were described.

In seven case series, the included patients had a diagnosis of primary NPC [28–30, 32–35]. In two of these studies, distant metastases were identified [28, 30]. In other four series [3, 5, 31, 37], the included patients had a diagnosis of splenic metastases; while splenic focal lesions were identified at imaging in another study [39]. Most cases of splenic metastases (*n* = 15) were detected by imaging techniques (US, CT, MRI, and PET/CT). In two cases [29, 31], the detection method was not clearly reported. Three cases were found at autopsy [5]. Another five cases were reported in a study that combined data from autopsy and splenectomy [3]. The histological confirmation of NPC metastases to the spleen was reported in only eight cases. For the other cases (*n* = 17), the NPC origin was reported according to the history of the primary tumor and imaging (Tables 1 and 2).

**Table 1:** Summary of the studies reporting on solitary splenic metastases from NPC

Year	First author	Type of study	Number and type of patients considered	Patients with solitary splenic metastases from NPC	Patient’s age, sex (M, F)	Comorbidity	Primary treatment for NPC	Disease-free survival after primary treatment	Methods of detection of splenic metastasis	Treatment for solitary splenic metastasis	Confirmatory histology of NPC splenic metastases	Overall survival (status at last follow-up)	Relapse after splenic surgery
2000	Lam & Tang [3]	CS	92 patients with splenic metastases	1 (1.1 %)	M	NS	NS	NS	NS	- Splenectomy	UCNT	NS	NS
2013	Suh et al. [38]	CR	1 non-keratinizing NPC	1	35 years old, M	EBV	- Neoadjuvant chemotherapy (cysplatin and 5-fluorouracil) - Left medial maxillectomy with left modified radical neck dissection - Adjuvant radiochemotherapy (cysplatin and 63 Gy)	7 months	FDG-PET/CT FNA biopsy	- Systemic chemotherapy (docetaxel and cysplatin) - Laparoscopic splenectomy	UCNT	5 months (alive)	Nil
2015	Abu-Zaid et al. [27]	CR	1 NPC	1	26 years old, M	NS	- Surgical resection - Adjuvant radiochemotherpay	6 years	CT FNA biopsy	- Splenectomy	UCNT	12 months (alive)	Nil
2015	Present study	CR	1 NPC	1	56 years old, M	Smoker, EBV	- Neoadjuvant chemotherapy (docetaxel, carboplatin, and 5-Fluorouracil) - Radiotherapy (70 Gy)	3 months	PET/CT	- Laparoscopic splenectomy	UCNT	4 months (alive)	Nil

*CR* case report, *CS* case series, *NPC* nasopharyngeal carcinoma, *UCNT* undifferentiated carcinoma of the nasopharyngeal type, *M* male, *F* female, *EBV* epstein-barr virus, *SCC* squamous cell carcinoma, *FNA* fine-needle aspiration, *CT* computed tomography, *MRI* magnetic resonance imaging, *PET* positron emission tomography, *NS* not specified

**Table 2:** Summary of the studies reporting on non-solitary (or not specified) splenic metastases from NPC

Year	First author	Type of study	Number and type of patients considered	Patients with splenic metastases from NPC	Methods of detection	Surgery performed	Confirmatory histology of NPC splenic metastases	Relapse and survival rates
1952	Abrams et al. [5]	CS	1000 patients with splenic metastases from carcinomas	3 (0.3 %)	Autopsy	NS	NS	NS
1989	Siniluoto et al. [37]	CS	31 patients with splenic metastases	1 (3.2 %)	US	NS	NS	NS
2000	Wan et al. [39]	CS	53 patients with focal splenic lesions	2 (3.7 %)	US	NS	NS	NS
2000	Lam & Tang [3]	CS	92 patients with splenic metastases	4 (4.3 %)	NS	NS	UCNT	NS
2001	Gacani et al. [30]	CS	65 patients with NPC and DM	1 (1.5 %)	US	NS	NS	DM within 24 months after therapy
2004	Cho et al. [29]	CS	31 patients with recurrent type 2 NPC	1 (3.2 %)	NS	NS	NS	NS
2010	Radhakrishnan et al. [36]	CR	1 pediatric patient with UCNT	1	FDG-PET/CT	NS	NS	Death after 90 days of chemotherapy
2010	Ng et al. [35]	CS	179 NPC patients at high risk of residual disease or with suspected recurrence	1 (0.5 %)	WB-MRI and FDG-PET/CT	NS	NS	NS
2011	Gatenby et al. [31]	CS	21 patients undergone splenectomies for DM	1 (4.7 %)	NS	Primary tumor, radical neck dissection and synchronous splenectomy	SCC	DFS: 1 year 10 month OS: 2 years 3 months
2013	Hsieh et al. [32]	CS	22 patients with non-keratinizing or undifferentiated NPC	1 (4.6 %)	US, CT or MRI	NS	NS	Median time to PD: 10 months; Median OS: 16 months
2015	Mak et al. [34]	CS	558 patients with NPC	1 (0.2 %)	CT and PET/CT	NS	NS	10.8–18.4 months before DF; Mean DSS in patients with DM: 31.2 months (95 % CI 20.9–41.6) Mean OS in patients with DM: 28.2 months (95 % CI 19.3–37.1)
2015	Ma et al. [33]	CS	2 pediatric patients with NPC	1	FDG-PET/CT	NS	NS	NS
2015	Al Tamimi et al. [28]	CS	352 patients with NPC and DM	4 (1.1 %)	FDG-PET/CT	NS	NS	NS

*CR* case report, *CS* case series, *NPC* nasopharyngeal carcinoma, *UCNT* undifferentiated carcinoma of the nasopharyngeal type, *SCC* squamous cell carcinoma, *DM* distant metastases, *NS* not specified, *CT* computed tomography, *MRI* magnetic resonance imaging, *PET* positron emission tomography, *US* ultrasounds, *FNA* fine-needle aspiration, *DF* distant failure, *DFS* disease-free survival, *DSS* disease-specific survival, *OS* overall survival, *PD* progressive disease

Solitary splenic metastases with histological confirmation were reported in three cases only [3, 27, 38] (Table 1). In particular, the case reported by Suh et al. [38] had a presentation similar to our patient. Indeed, the authors described the case of a young male patient with a history of NPC who had a disease-free survival for 7 months after surgery and chemoradiation therapy. The patient was asymptomatic when a hypermetabolic splenic lesion was detected at the PET/CT scan. A percutaneous biopsy confirmed the metastatic involvement of the spleen and a laparoscopic splenectomy was performed. No recurrence was observed after 5 months of follow-up. Similarly, Abu-Zaid et al. [27] reported the case of a young man with a history of NPC who experienced referred pain in the upper abdominal quadrant. A splenic lesion was detected by means of a CT scan, and an ultrasound-guided biopsy confirmed a NPC metastasis. In all cases, the confirmatory histology showed an UCNT type of NPC solitary splenic metastasis, and all patients underwent successful splenectomy. No study reported relapse or recurrence after the treatment of splenic metastasis.

#### Prevalence of NPC metastases to the spleen

The overall number of patients analyzed in the studies selected for this systematic review was 2409, including 1212 patients with a diagnosis of NPC [27–30, 32–36, 38] and 1197 patients with a diagnosis of splenic metastases from several primary tumors or splenic focal lesions [28, 33, 35, 37, 39]. Of the 1212 patients with a diagnosis of NPC, 417 had also distant metastases. Splenic metastases were detected in 1.07 % of all NPC patients (13/1212) and in 1.19 % of patients with NPC and distant metastases (5/417). Moreover, NPC represented 1 % of the primary sources among patients with a diagnosis of splenic metastases secondary to all solid tumors (12/1197).

#### Study quality assessment

Two reviewers (PG and FB) scored the methodological qualities of the included studies according to the criteria described above. No RCT was found. The studies were case reports or case series with different methods and aims. The GRADE system was used to enable a consistent judgment of the quality of the available evidence included in this systematic review, and the studies retrieved were judged as having evidence of very low quality. Of note, the majority of the studies were retrospective, which, by definition, are susceptible to major selection bias, as well as misclassification, detection, or information bias due to the unknown accuracy of record keeping [40]. Moreover, other specific sources of bias (e.g., attrition and reporting bias) cannot be ruled out. The heterogeneous features of the studies evaluated, the low number of pertinent articles found and the lack of specific studies in the literature restrict the possibility of large remarks and represent the main limitations of the present systematic review.

## Discussion

The present study describes a rare case of a patient who underwent splenectomy for a solitary splenic metastasis from UCNT. Moreover, the systematic review found 25 cases of splenic metastases from NPC reported in the literature, with solitary metastases reported in only three of these cases. Based on the available literature, the estimated rate of metastases to the spleen in patients with a diagnosis of NPC is approximately 1 %. Moreover, NPC represents the 1 % of all sources of spleen metastases among metastatic solid cancers.

The very low number of splenic metastases from NPC reported in the literature defines this event as rare. The most recent study on this topic was published in 2007 and reported 93 well-documented cases of solitary splenic metastases [1]. The primary source was a gynecologic cancer in 29 % of these cases (19 % ovarian and 7 % endometrial) and colorectal cancer in 21 %. Several other primary sites, such as the lung, esophagus, stomach, kidney, breast, prostate and skin, were described, but no case of metastases from NPC was reported.

In most of the cases, splenic metastases are diagnosed incidentally in asymptomatic patients. However, splenic metastases, especially the isolated ones, may also occur in association with non-specific clinical manifestations, such as fatigue, weight loss and fever; anemia or thrombocytopenia caused by hypersplenism; pain in the left upper abdominal quadrant; splenomegaly or spontaneous splenic rupture [3, 10, 14, 41–45]. Symptomatic lesions are more frequently reported in women and in younger patients, and the mean maximum size of the lesions in these patients is usually larger than in asymptomatic patients [3]. Moreover, the presence of splenic metastatic foci might also be associated with an increase in serum tumor markers, which might precede the imaging detection of splenic lesions by years [9].

Several authors reported that the increased use of imaging and PET scan, the close follow-up and the prolonged survival favored an increasing detection of metastases to the spleen [1, 9, 46]. Most often, splenic metastases are diagnosed by means of ultrasonography or CT, but MRI can also be used to study splenic focal lesions [9]. However, the differentiation between benign and malignant splenic focal lesions can be difficult using these techniques, and an 18F-FDG scan is often performed [1, 9, 46]. A positive history of cancer appeared as the only independent predictive factor for malignancy of a splenic lesion [9], and this is consistent with our case report and with the current literature [3, 10, 14, 41–45] Generally, in patients with a history of malignancy, a solitary splenic lesion should be first considered as a metastases [10]. In these patients, a histologic diagnosis should be achieved by percutaneous biopsy or splenectomy [1, 47–49]. According to several studies, imaging-guided percutaneous biopsy of suspicious splenic lesions is relatively safe and accurate [9, 11], with a diagnostic yield and accuracy of 90–92 and 95 %, respectively, in front of a 2 % rate of major complications [1, 9]. However, splenectomy is much more common in clinical practice, mainly because of the hemorrhagic risk [1], showing a diagnostic yield of 95 % and representing at the same time a therapeutic procedure [11]. Splenectomy has been reported for both synchronous or metachronous metastases [31, 48–51], and some authors considered the surgical treatment of isolated splenic metachronous metastases as effective as for hepatic and pulmonary secondary lesions in the control of the neoplastic disease [2, 48, 51–53]. However, splenectomy for solitary metastases also represents a therapeutic challenge because of its uncertain impact on the patient’s prognosis. Indeed, splenic involvement is generally linked to a widespread disease [7, 48], and a survival inferior than 30 days was reported in case of infarction in a metastatic spleen [54].

To assess the role of surgery in the treatment of isolated splenic metastases, Piardi et al. [48] reported 28 cases of splenectomy for isolated metastases to the spleen, which in most cases involved single lesions. No increase complication rate or mortality was reported, with a disease-free and an overall survival rates ranging between 3 and 31 months and between 3 and 96 months, respectively. The authors concluded that, although prognosis is always linked to the primary tumor stage, splenectomy can be justified because it might avoid complications related to a progressive increase in the metastatic volume and an infiltrative spread involving surrounding organs and tissues, especially diaphragm, and abscess formation [10, 55]. Furthermore, splenectomy could also be justified as a debulking procedure before chemotherapy [55–57], it could result in longer survival [51, 57], and it should be performed shortly after the detection of the splenic metastasis [57].

Both laparoscopic and open splenectomy can be performed. When operating for malignancies, the open approach is usually preferred because it could provide an easier access to other areas if needed [31]. However, several studies considered laparoscopic splenectomy a highly reliable procedure that can be performed safely [58]. In our case report, the lesion was well-delimited with no invasion of surrounding tissues, and no difficulties were encountered in performing a laparoscopic splenectomy.

To date, it is difficult to predict the clinical behavior of solitary splenic metastases because its occurrence is rare, and the literature on this topic contains mostly case reports with short follow-up periods. Cancer cells already implanted in the splenic parenchyma might not be detected at the time of primary diagnosis by conventional methods, and the hostile splenic environment might not facilitate the growth of micrometastatic foci. This might explain the contrast between the prevalence of splenic micro-metastases at autopsy in patients with multivisceral cancer and the rarity of clinically detectable lesions. Therefore, splenic metastases might result from the growth of early blood-borne disseminated cancer cells within the spleen after a certain period of clinical latency [1, 10], which sometimes is very long (up to 7 [3] or 11 years [59]). This might also explain the long-term remission achieved in some patients treated with splenectomy alone [3, 10, 13, 48, 57] supporting that isolated splenic metastases are not necessarily the precursor sign of active metastatic cancer in the terminal stage.

The anatomic extent of the metastasis is closely associated with the prognosis of the patients with metastatic NPC [60]. A single metastatic lesion in an isolated location (organ or site) is reported to be associated with prolonged survival compared to multiple metastatic lesions in a single or multiple locations [61, 62]. A growing body of evidence shows that long-term survival could be achieved for selective NPC patients with limited metastatic lesions by a combination of systemic and local therapies [63, 64]. The reported overall survival of NPC patients after the detection of distant metastases ranged from 22 to 120 months [60, 61, 65]. Furthermore, the distant failure hazard is estimated to decrease by 19 % for each year the latency [66].

The great heterogeneity encountered in the studies analyzed in the present systematic review, particularly the different study designs, aims and methods, represents the main limitation of the present study. Most of the studies were published between 2000 and 2015, although two were dated in the 1980s and 1950s. This very large time frame may also impact on the heterogeneity of diagnostic and therapeutic protocols changed and improved over the years. Important data, such as survival rates, were not always specific for NPC cases of splenic metastases. Autopsy series hamper the possibility to remark on the clinical aspects and prognosis. However, despite these limitations mainly inherent in the currently available studies, the present study is the first one to systematically review the literature on splenic metastases from NPC, and it can be useful to build a general framework regarding splenic metastases, thus providing useful information to guide clinical practice. Further and more specific studies are needed to better assess the incidence of splenic involvement in patients with NPC, and to evaluate the impact of splenectomy in cases of metastases to the spleen.

## Conclusions

According to the literature reviewed, splenic metastasis are rare but should be considered for patients with a history of NPC and diagnosis of splenic lesions at imaging. There is no evidence supporting a negative impact of splenectomy in patients with isolated splenic metastases from NPC.

## Abbreviations

CR, complete remission; CT, computed tomography; ENT, ear, nose, throat; NPC, nasopharyngeal carcinoma; PET, positron emission tomography; RCT, randomized controlled trail; SUV, standardized uptake value; UCNT, undifferentiated carcinoma of nasopharyngeal type; US, ultrasound
